# Near-infrared fundus autoflorescence imaging in solar retinopathy

**DOI:** 10.3205/oc000056

**Published:** 2017-03-03

**Authors:** Maciej Czepita, Anna Machalińska, Damian Czepita

**Affiliations:** 1 Department of Pathology, Pomeranian Medical University, Szczecin, Poland; 2 Department of Ophthalmology, Pomeranian Medical University, Szczecin, Poland

**Keywords:** solar retinopathy, near-infrared fundus autofluorescence, fundus autofluorescence

## Abstract

Solar retinopathy is a rare clinical entity caused by photochemical damage to the retinal pigment epithelium layer and photoreceptors of the fovea. Here we describe a case of a 33-year-old female patient diagnosed by near-infrared fundus autofluorescence imaging for signs of damage to the melanosomes of the retinal pigment epithelium of the fovea. The patient was advised to discontinue looking at the sun with the naked eye.

## Introduction

Solar retinopathy is a rare ocular disease. It is mostly due to unprotected observing of the sun usually during solar eclipses [1]. Currently the imaging modalities most often used in the diagnosis of solar retinopathy are optical coherence tomography (OCT) and short-wavelength fundus autofluorescence (SW-FAF). Here we report for the first time to our knowledge near-infrared fundus autofluorescence (NIA) findings using the Heidelberg Spectralis HRA+OCT in a patient with solar retinopathy.

## Case description

**Figure 1:**
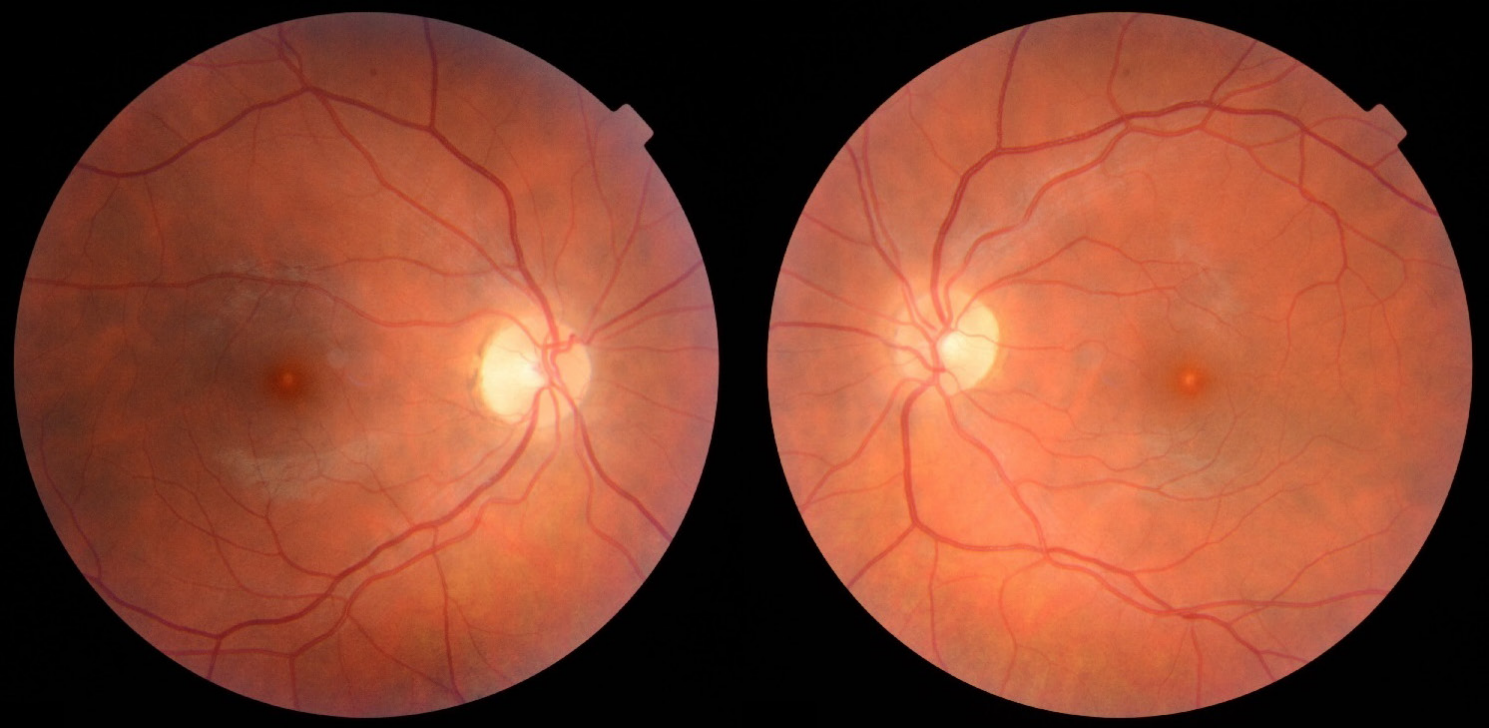
Color fundus image of both eyes that show white discolorations in the fovea

**Figure 2:**
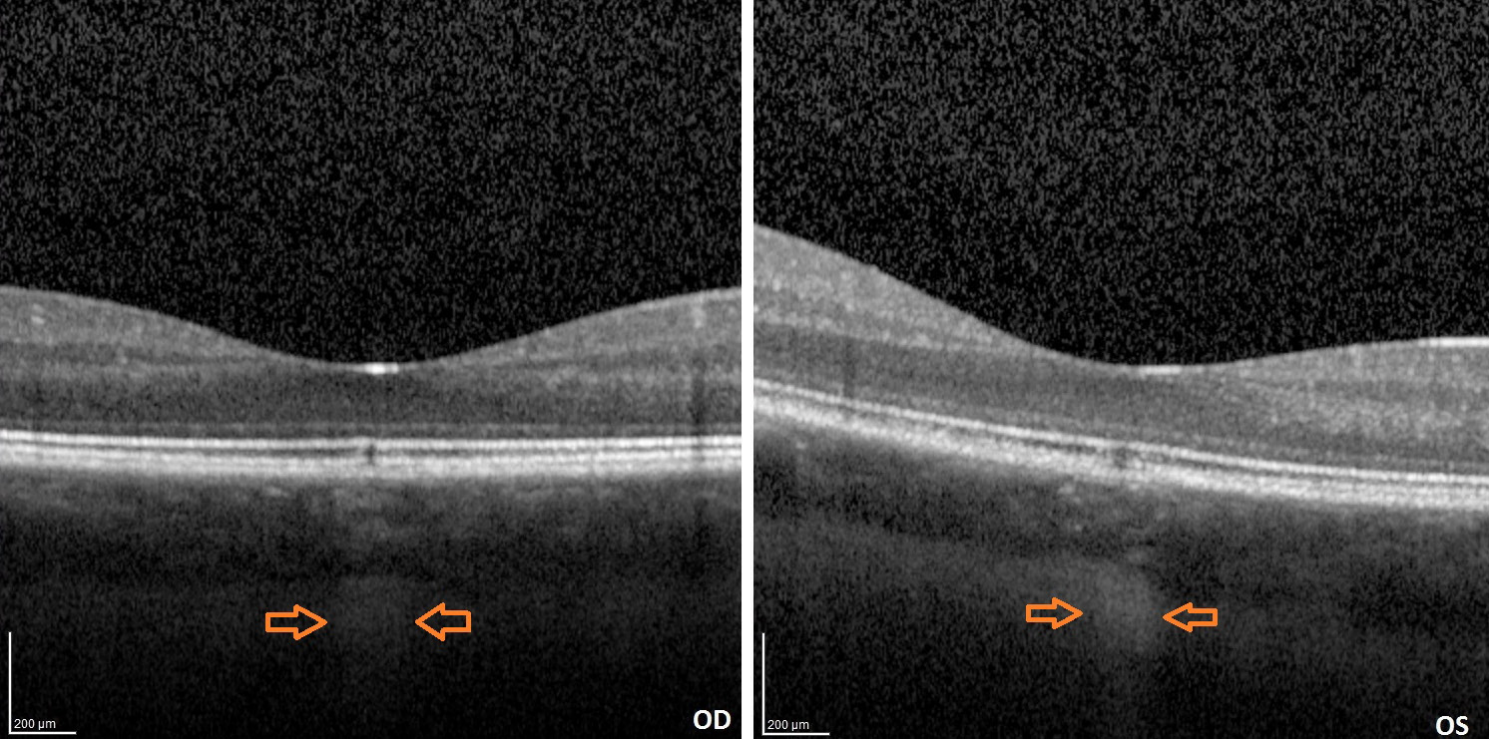
SD-OCT image of both eyes: orange arrows point to streaks of increased reflectivity at the sclera and above it in the choroid.

**Figure 3:**
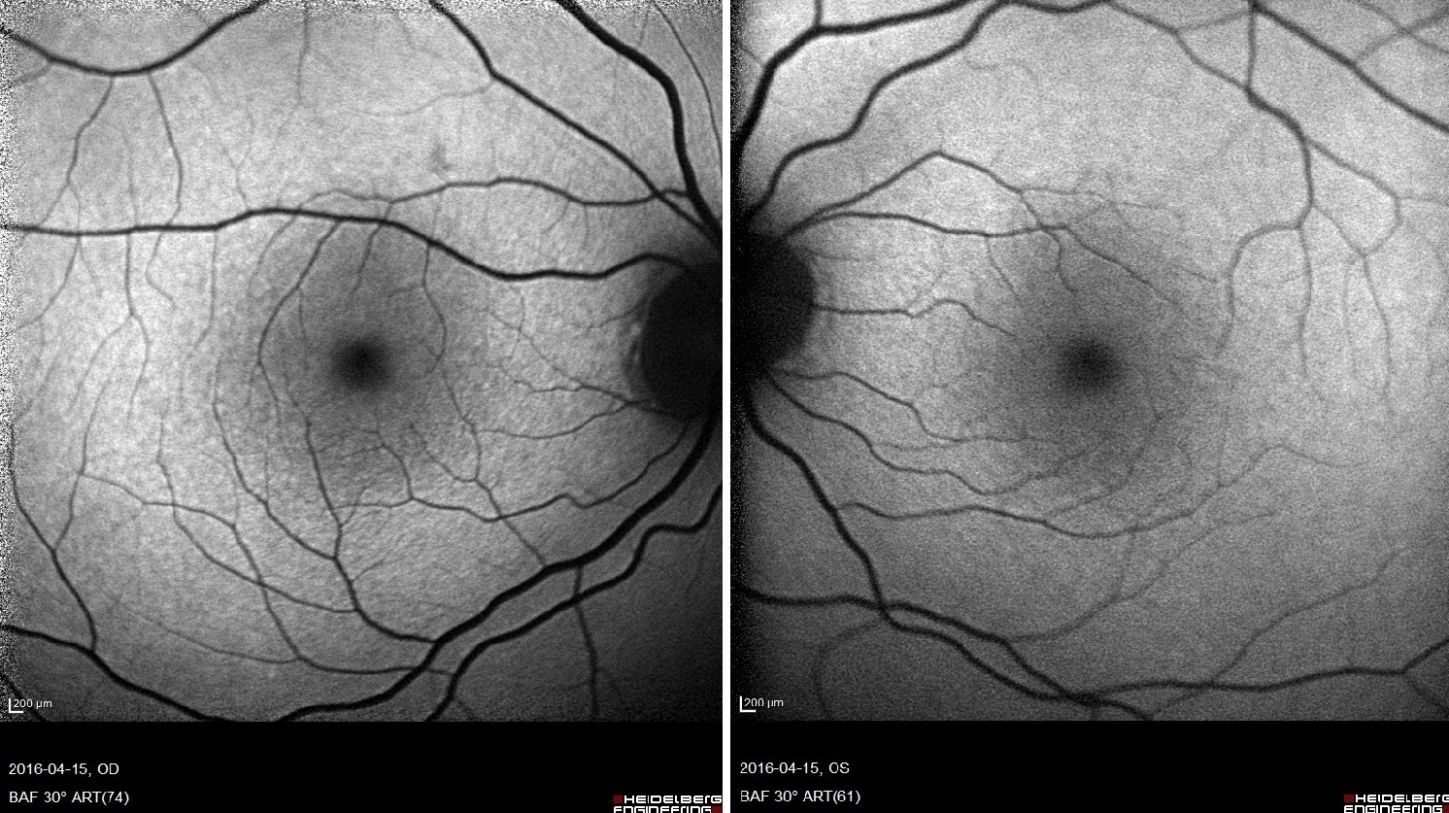
Short-wavelength fundus autofluorescence (SW-FAF) images of both eyes: no changes visible

**Figure 4:**
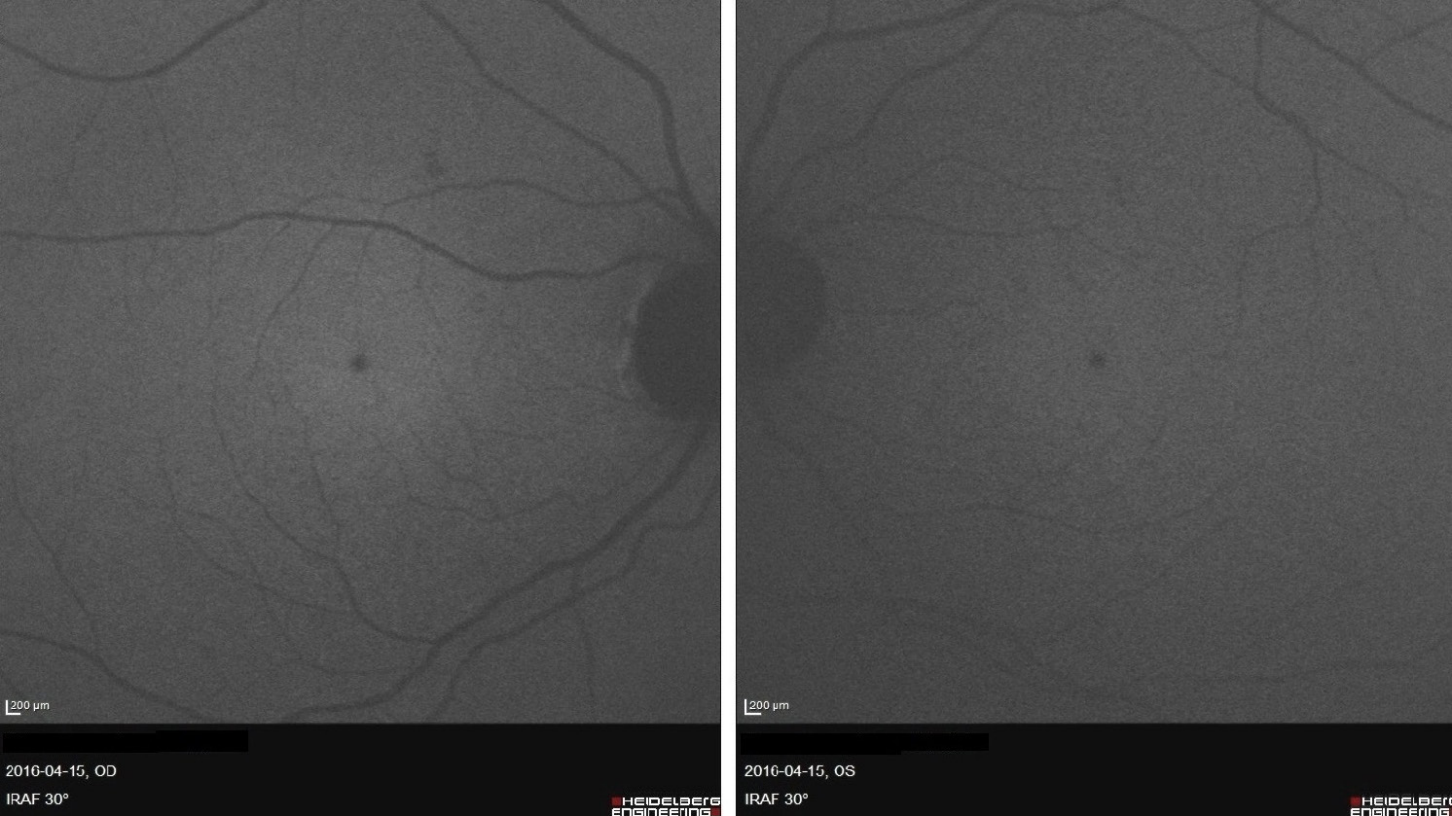
Near-infrared fundus autofluorescence images (NIA) of both eyes: clearly visible hypoautofluorescent spots in the fovea

A 33-year-old female patient of Latvian descent reported with a history of decreased bilateral visual acuity. Initially the patient’s history was unremarkable, but during her second visit she acknowledged that for spiritual purposes she had been observing the sun with the naked eye regularly for many minutes over the past 8 years. Her uncorrected distance visual acuity (UCVA) was 0,9 in both eyes. A color fundus image disclosed a small round whitish discoloration in the fovea of both eyes (Figure 1 (Fig. 1)). A discrete discontinuity in the inner and outer segment junction (IS/OS), cone outer segment layer (COST), and retinal pigment epithelium layer (RPE) in the fovea of both eyes was noted on SD-OCT along with a streak of increased reflectivity in the choroid and sclera corresponding in size and location to the whitish discoloration on the color fundus image probably caused by focal RPE atrophy (Figure 2 (Fig. 2)). No changes were found on short-wavelength fundus autofluorescence (SW-FAF) (Figure 3 (Fig. 3)). However, on near-infrared fundus autofluorescence (NIA) small spots of hypoautofluorescence in both maculae corresponding in location to the white discoloration in the color fundus image and the IS/OS to RPE layer retinal layer disruption on SD-OCT were found (Figure 4 (Fig. 4)). The hypoautofluorescent spots were also similar in size to the white discoloration in the color fundus image. The anterior segment examination was normal in both eyes. Based on these results we diagnosed the patient with solar retinopathy and advised her to discontinue looking at the sun with the naked eye.

## Discussion

Near-infrared fundus autofluorescence (NIA) is a relatively new and emerging ophthalmic imaging technique. The source of autofluorescence in the near-infrared seems to originate from melanin in the RPE and to a varying degree from the melanin in the choroidal layers [2]. It has been used in various retinal disorders such as age-related macular degeneration (AMD) [3], central serous chorioretinopathy (CSCR) [4] or multiple evanescent white dot syndrome (MEWDS) [5] yielding interesting new insights. To our knowledge, this is the first published case report of near-infrared fundus autofluorescence findings in solar retinopathy. However, related work was previously carried out by Neri et al. [6] involving a 28-year-old female patient diagnosed with photic maculopathy resulting from exposure to a high power arc flood lamp. Although no changes were detected on the initial examination on short wave fundus autofluorescence, near-infrared fundus autofluorescence did show a prominent spot of hypoautofluorescence in the foveola of both maculae similar to the ones observed in our patient. Our findings are consistent with the current understanding of the pathogenesis of solar retinopathy in which the primary lesion seems to occur in the melanosome – containing RPE layer followed by subsequent photoreceptor damage, likely secondary to disruption of the supportive RPE [7]. This was clearly demonstrated in our patient as a well defined spot of hypoautofluorescence in the foveola of both maculae. Further investigations are warranted in order to conclude the reliability and reproducibility of our findings. Overall, the use of near-infrared fundus autofluorescence imaging in patients with suspected solar retinopathy should be considered. When analyzed with results from OCT and SW-FAF it can greatly help in making the correct diagnosis.

## Notes

### Competing interests

The authors declare that they have no competing interests.

### Informed consent

Informed consent was obtained from the patient. The study was approved by the Bioethics Committee of the Pomeranian Medical University in Szczecin, Poland.
